# In‐depth echocardiographic analysis of left atrial function in healthy adults using speckle tracking echocardiography and volumetric analysis

**DOI:** 10.1111/echo.14174

**Published:** 2018-10-30

**Authors:** Roderick W. J. van Grootel, Mihai Strachinaru, Myrthe E. Menting, Jackie McGhie, Jolien W. Roos‐Hesselink, Annemien E. van den Bosch

**Affiliations:** ^1^ Department of Cardiology Erasmus MC Rotterdam The Netherlands; ^2^ Department of Radiology Erasmus MC Rotterdam The Netherlands

**Keywords:** atrial function, left atrium, myocardial function, reference values, speckle tracking echocardiography

## Abstract

**Purpose:**

Left atrial (LA) dilatation is predictive for complications in a multitude of cardiac diseases; therefore, adequate assessment is essential. Technological advances have made it possible to quantify LA function with Speckle Tracking Echocardiography (STE); however, there are currently no recommendations for normal values with regard to LA function. We aimed to assess LA myocardial and volumetric function in a healthy cohort and investigate correlations with baseline characteristics.

**Methods:**

This prospective cohort study included 147 (aged 20–72) healthy individuals and assessed LA volumetric function using maximum, minimum and pre‐a‐wave volumes and myocardial function using reservoir function using peak strain in LA relaxation (LA‐strain), conduit function using peak strain rate in early LA contraction (LA‐SRe) and pump function using peak strain rate in late LA contraction (LA‐SRa).

**Results:**

Mean LA‐strain was 39.7 ± 6.2%, LA‐SRe −2.78 ± 0.62 s^−1^ and LA‐SRa −2.56 ± 0.62 s^−1^. Subjects were divided into 5 age decades (each 50% female). LA‐strain and LA‐SRe were lower in the oldest groups, whereas LA‐SRa was higher. LA‐SRa was higher in males(−2.69 ± 0.68 s^−1^ vs −2.42 ± 0.52 s^−1^). Age‐specific values are provided. Age proved to be an independent predictor for LA‐SRa after correction for blood pressure and heart rate. LA expansion index and passive emptying fraction decreased with age, while active emptying fraction increased with age. LA maximum volume did not increase with age.

**Conclusion:**

This study provides normal values for the three phasic functions of the LA, assessed with STE and volumetric function. Our results suggest the need for age‐specific reference ranges, and normal values for this cohort have been calculated.

## 1 INTRODUCTION

Assessment of the left atrium (LA) is gaining increased attention as it reflects the severity and chronicity of many different conditions and is associated with significant morbidity and mortality.^1^

In the absence of valvular disease, LA volume reflects the presence of elevated left ventricular (LV) diastolic pressure and dysfunction.^2^ LA maximum volume is the most often described parameter, but LA phasic function could be a more sensitive measure in patients with heart failure, valvular disease, and atrial fibrillation. LA function can be assessed by volumetric measurements and includes reservoir, conduit, and pump function which can be expressed as absolute volumes or fractions. Recently speckle tracking echocardiography (STE) has been validated for LA measurements^3^; LA strain and strain rate can be measured which reflect LA myocardial function without the need for geometrical assumptions.

The clinical value of LA volumetric and myocardial function has not been translated into recommendations to be used in clinical practice. This is in part because solid reference ranges have not been established, neither for volumetric measurements^4^, ^5^, ^6^ nor for strain measurements.^7^, ^8^, ^9^, ^10^, ^11^, ^12^

Therefore this study aims to provide reference ranges for LA myocardial and volumetric function in healthy adults and investigates the impact of age, sex, and BSA.

## 2 METHODS

### 2.1 Study design and population

Healthy volunteers were enrolled in 2014–2015 for this prospective cross‐sectional study and stratified into 5 age groups: 20–29, 30–39, 40–49, 50–59, 60–72 years (n ≥ 28 for each group, each 50% female). Details have been published earlier.^13^ Briefly, subjects were recruited via advertisement and underwent a questionnaire regarding medical history and current health status, physical examination, venous blood sampling, 12‐lead ECG, and an echocardiogram. Subjects were excluded if one or more of the following criteria were present: (prior) cardiovascular disease, systemic disease, the finding of cardiac abnormalities during the examination (including any valvular abnormalities) or risk factors including hypertension (cutoff values: 140/80 mm Hg), diabetes mellitus, impaired renal function or hypercholesterolemia. In case of elevated blood pressures, follow‐up measurements were performed by the general practitioner to confirm this. If follow‐up revealed normal blood pressures, the subject was included. Reasons for exclusion due to abnormalities on ECG were conduction disorders: Atrial fibrillation, right or left bundle branch block, prolonged PR interval, and prolonged QRS. Professional athletes, people who were morbidly obese (BMI > 40 kg/m^2^), having breast implants or pregnant were excluded. This study was carried out according to the principles of the Declaration of Helsinki and approved by the local ethics committee. Written informed consent was obtained from every participant.

### 2.2 Echocardiographic image acquisition

Echocardiographic studies were performed by one of two experienced sonographers. Two‐dimensional grayscale harmonic images were obtained in the left lateral decubitus position using a iE33 or EPIQ7 ultrasound system (Philips Medical Systems, Best, The Netherlands) equipped with a transthoracic broadband X5‐1 matrix transducer (composed of 3040 elements with 1–5 MHz). The LA was acquired in dedicated apical four‐ and two‐chamber views with frame rates ≥ 50 frames/s.^14^ At least 2 consecutive heartbeats were recorded.

### 2.3 Volumetric analysis

**Figure 1:**
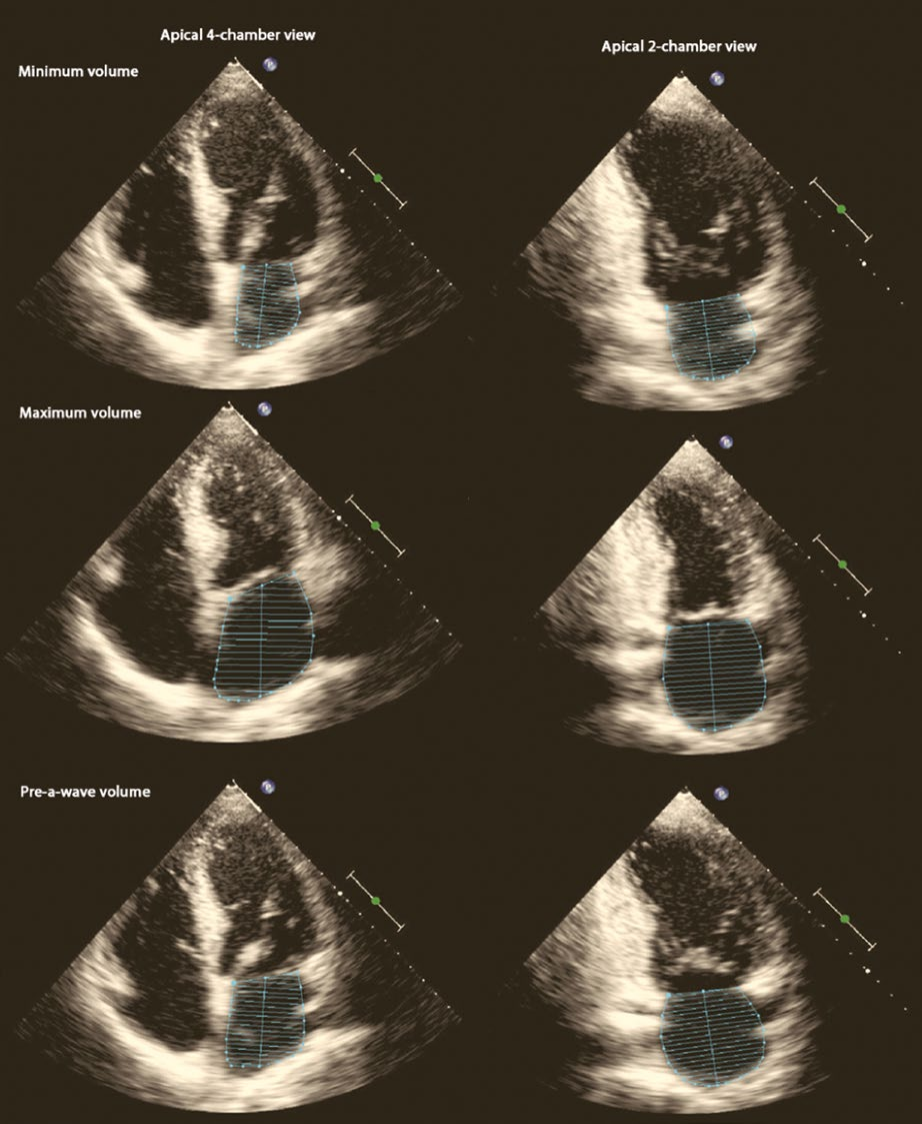
Example of the volumetric measurements using the method‐of‐disk summation technique in dedicated apical four‐ and two‐chamber views. From top to bottom: the left atrial minimum, maximum, and pre‐a‐wave volume

In order to assess LA maximum volume, the revised recommendations for cardiac chamber quantification were used.^1^ LA minimum volume (measured at mitral valve closure) and pre‐a‐wave volume (one frame before atrial contraction starts) were measured using the biplane method‐of‐disk summation technique (Figure 1) and the area‐length method. All measurements were performed with Xcelera (Philips Medical Systems). Using the above volumes, LA function can be assessed as follows:

Left atrial reservoir function:

- Left atrial total emptying volume (TEV) = LA maximum volume–LA minimum volume.
- Left atrial total emptying fraction = TEV/LA maximum volume.
- Left atrial expansion index = TEV/LA minimum volume.

Left atrial conduit function:

- Left atrial passive emptying volume (PEV) = LA maximum volume–LA pre‐a‐wave volume.
- Left atrial passive emptying fraction = PEV/LA maximum volume.

Left atrial pump function:

- Left atrial active emptying volume (AEV) = LA pre‐a‐wave volume–LA minimum volume.
- Left atrial active emptying fraction = AEV/LA pre‐a‐wave volume.

All reported volumes are indexed for BSA. Since the Dutch population is the tallest in the world,^15^ we indexed for an allometric function of height^2.7^.^16^ LV diastolic function was assessed according to the EAE‐ASE recommendations for diastolic function.^17^

### 2.4 Speckle tracking analysis

**Figure 2:**
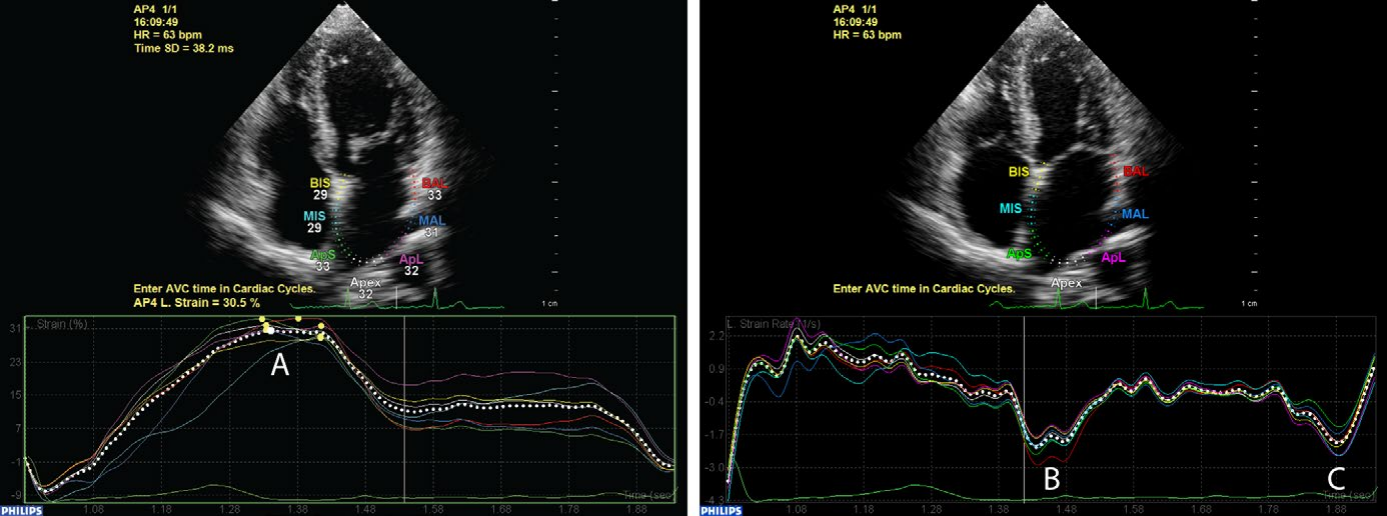
Example of left atrial (LA)‐strain measurement in a apical four‐chamber view. LA‐strain(A) is measured as the maximum strain value during atrial diastole. Conduit (B) and pump (C) function are measured using strain rate

Offline analysis was performed using QLAB10 (Philips Medical Systems). LA myocardial function was assessed according to an earlier published guideline and a recent validation study,^18^, ^19^ using the apical four‐ and two‐chamber views and the R‐wave as reference point. LA reservoir function can be expressed as peak strain (LA‐strain) and LA conduit and pump function with LA strain rate. The negative peak in early diastole represents LA conduit function (LA‐SRe) and the negative peak in late diastole represents LA pump function (LA‐SRa) (Figure 2).

### 2.5 Statistical analysis

Normal distribution was checked using histograms and Shapiro‐Wilk tests. Depending on data distribution, continuous data are presented as mean ± standard deviation (SD) or median with first‐third quartile. Categorical data are presented as frequencies and percentages. Student's *t*‐test, the Mann–Whitney *U* test, chi‐square test or Fisher's exact test was used when appropriate. Correlations between LA measurements and baseline characteristics were assessed using the Pearson correlation test. When a variable was statistically significant and did not show collinearity with another variable, they were included in a multivariable linear regression model. In case of collinearity, the one with the strongest correlation was selected. Statistical analysis was done with the Statistical Package for Social Sciences version 21 (IBM DPDD Statistics for Windows, Armonk, NY, USA). A *P*‐value of ≤0.05 (two‐sided) was considered statistically significant.

Interobserver (RG, MS) agreement was assessed for LA volumetric and strain parameters using Bland–Altman plots in a sample of 30 random subjects.^20^ Measurements were done while being blinded for the other measurement approximately 1 month later. Agreement between two measurements was determined as the mean of the difference ± 1.96 SD.

## 3 RESULTS

Out of the 155 eligible subjects, 147 subjects were included (median age 43.8 [32.7–56.2], 50% female) into 5 age groups (n ≥ 28 per group). In total, 8 subjects were excluded: 2 due to having breast implants, 2 subjects had valvular pathology, 1 had a surgically closed ductus, 1 had hypertension, 1 with morbid obesity, and 1 with a right bundle branch block. Table 1 shows the baseline characteristics of the study population.

**Table 1:** Baseline table

	Total n = 147	Male n = 73	Female n = 74	*P*‐value
Age (years)	44.6 ± 13.8	44.0 ± 13.7	45.3 ± 13.8	ns
Height (cm)	175 ± 9	181 ± 7	169 ± 6	**<0.001**
Weight (kg)	74.6 ± 12.8	82.4 ± 11.2	66.9 ± 9.0	**<0.001**
Body mass index (kg/m²)	24.4 ± 3.3	25.2 ± 3.3	23.6 ± 3.0	**0.002**
Body surface area (m²)	1.89 ± 0.19	2.03 ± 0.15	1.76 ± 0.12	**<0.001**
Systolic blood pressure (mm Hg)	127 ± 15	131 ± 16	123 ± 12	**0.001**
Diastolic blood pressure (mm Hg)	80 ± 9	82 ± 9	77 ± 9	**<0.001**
Creatinine (μmol/L)	78 ± 12	85 ± 10	71 ± 10	**<0.001**

Bold means statistically significant difference between both groups.

### 3.1 LA volumetric function

Feasibility for volumetric measurements was good, ranging from 92.5% to 95.9% (Table 2). LA volumes were indexed for BSA (Table 2), and an additional analysis was performed with height indexed parameters (Table 3). Changes in volumes can be seen between the age groups regardless of the indexation method. LA minimum and pre‐a‐wave volumes increased with each age decade. With regard to function, LA reservoir and conduit function decreased while pump function increased with age (Table 4).

**Table 2:** Left atrial echocardiographic volumes indexed for BSA per age decade

	Feasibility (%)	Entire study	20–29 y	30–39 y	40–49 y	50–59 y	60–72 y	*r*	*P*‐value
		n = 147	n = 32	n = 28	n = 28	n = 31	n = 28		
Method‐of‐disk summation technique									
LA maximum volume (mL/m²)	n = 136 (92.5%)	28.8 ± 7.2	27.8 ± 5.7	28.1 ± 6.6	29.0 ± 9.2	29.4 ± 5.5	30.0 ± 9.1	ns	ns
LA minimum volume (mL/m²)	n = 141 (95.9%)	10.1 ± 3.7	8.7 ± 2.5	9.4 ± 2.8	10.8 ± 4.0	10.4 ± 3.2	11.9 ± 5.2	0.252	**0.003**
LA pre‐a‐wave volume (mL/m²)	n = 141 (95.9%)	18.1 ± 5.5	14.5 ± 3.8	16.4 ± 3.9	18.8 ± 5.9	19.4 ± 4.0	21.9 ± 6.9	0.437	**<0.001**
Area‐length method									
LA maximum volume (mL/m²)	n = 136 (92.5%)	31.0 ± 7.5	29.9 ± 6.0	30.2 ± 6.7	31.1 ± 9.4	31.7 ± 6.1	32.3 ± 9.6	ns	ns
LA minimum volume (mL/m²)	n = 141 (95.9%)	10.9 ± 3.9	9.4 ± 2.8	10.0 ± 3.1	11.5 ± 4.2	11.3 ± 3.3	13.0 ± 5.4	0.273	**0.001**
LA pre‐a‐wave volume (mL/m²)	n = 141 (95.9%)	19.4 ± 5.7	15.8 ± 3.9	17.5 ± 3.9	19.9 ± 6.1	21.0 ± 4.2	23.7 ± 7.1	0.463	**<0.001**

Correlation with age and corresponding *P*‐value are reported.

Bold means statistically significant correlation with age as a continuous variable.

**Table 3:** Left atrial echocardiographic volumes indexed for an allometric function of height^2.7^

	Entire study	20–29 y	30–39 y	40–49 y	50–59 y	60–72 y	*r*	*P*‐value
	n = 147	n = 32	n = 28	n = 28	n = 31	n = 28		
LA maximum volume (mL/m^2.7^)	12.2 ± 3.4	11.2 ± 2.5	11.7 ± 2.7	12.2 ± 4.4	12.6 ± 2.7	13.1 ± 4.4	0.202	**0.018**
LA minimum volume (mL/m^2.7^)	4.3 ± 1.7	3.5 ± 1.0	4.0 ± 1.1	4.5 ± 1.9	4.4 ± 1.3	5.2 ± 2.5	0.307	**<0.001**
LA pre‐a‐wave volume (mL/m^2.7^)	7.7 ± 2.6	5.9 ± 1.6	6.9 ± 1.5	7.9 ± 2.7	8.3 ± 1.9	9.5 ± 3.4	0.474	**<0.001**

Values are presented per age group and the correlation with age and corresponding *P*‐value are reported.

Bold means statistically significant correlation with age as a continuous variable.

**Table 4:** Left atrial function per age decade

	Feasibility (%)	Entire study	20–29 y	30–39 y	40–49 y	50–59 y	60–72 y	*r*	*P*‐value
		n = 147	n = 32	n = 28	n = 28	n = 31	n = 28		
LA volumetric function	n = 136 (92.5%)								
LA reservoir function (%)	Total emptying volume	18.7 ± 4.8	19.2 ± 4.7	18.7 ± 4.5	18.3 ± 6.3	19.0 ± 4.1	18.1 ± 4.7	ns	ns
	Expansion index	201.2 ± 71.4	238.5 ± 83.5	206.2 ± 46.2	184.2 ± 70.8	199.4 ± 77.2	169.3 ± 51.8	−0.262	**0.002**
	Total emptying fraction	65.9 ± 7.7	68.8 ± 7.3	66.6 ± 5.0	62.9 ± 8.5	64.8 ± 7.8	61.5 ± 7.4	−0.279	**0.001**
LA conduit function (%)	Passive emptying volume	10.8 ± 4.2	13.3 ± 3.1	11.7 ± 4.0	10.3 ± 4.6	10.0 ± 3.6	8.1 ± 3.9	−0.399	**<0.001**
	Passive emptying fraction	37.1 ± 11.3	47.9 ± 6.9	40.8 ± 9.4	34.7 ± 8.7	33.8 ± 9.4	26.6 ± 9.4	−0.613	**<0.001**
LA pump function (%)	Active emptying volume	8.0 ± 2.9	5.9 ± 2.3	7.0 ± 1.8	8.0 ± 3.0	9.0 ± 2.6	10.0 ± 2.8	0.512	**<0.001**
	Active emptying fraction	44.2 ± 10.0	40.3 ± 10.5	43.1 ± 7.5	43.3 ± 9.8	46.7 ± 10.2	47.1 ± 10.3	0.281	**0.001**
LA myocardial deformation analysis									
LA‐strain (%)	n = 118 (80.3%)	39.6 ± 6.3	41.7 ± 6.5	40.4 ± 5.3	39.1 ± 5.8	38.7 ± 6.9	37.1 ± 6.3	−0.227	**0.014**
LA‐Sre (s^−1^)	n = 115 (78.2%)	−2.76 ± 0.63	−3.29 ± 0.54	−3.06 ± 0.32	−2.77 ± 0.38	−2.23 ± 0.45	−2.16 ± 0.45	−0.715	**<0.001**
LA‐Sra (s^−1^)	n = 118 (80.3%)	−2.57 ± 0.62	−2.33 ± 0.52	−2.35 ± 0.40	−2.65 ± 0.55	−2.83 ± 0.80	−2.81 ± 0.63	0.348	**<0.001**
LV function									
E‐wave (m/s)		0.69 ± 0.16	0.79 ± 0.15	0.75 ± 0.16	0.66 ± 0.15	0.65 ± 0.11	0.59 ± 0.13	−0.457	**<0.001**
A‐wave (m/s)		0.49 ± 0.15	0.38 ± 0.14	0.43 ± 0.09	0.47 ± 0.10	0.57 ± 0.11	0.62 ± 0.17	0.582	**<0.001**
E/A‐ratio		1.6 ± 0.7	2.3 ± 0.8	1.8 ± 0.4	1.4 ± 0.4	1.2 ± 0.3	1.0 ± 0.3	−0.68	**<0.001**
Deceleration time (ms)		190 ± 41	178 ± 28	181 ± 32	185 ± 29	195 ± 32	216 ± 64	0.313	**<0.001**
E′ (LV septum) (cm/s)		9.5 ± 2.6	12.5 ± 1.8	10.4 ± 1.6	9.2 ± 1.6	8.2 ± 1.8	6.9 ± 1.7	−0.756	**<0.001**
E/E′‐ratio		7.6 ± 1.9	6.5 ± 0.2	7.3 ± 1.5	7.3 ± 1.7	8.1 ± 1.4	9.1 ± 2.4	0.472	**<0.001**
LV ejection fraction (%)		60 ± 5	60 ± 3.6	61 ± 5	59 ± 5	62 ± 5	59 ± 5	ns	ns

The upper part of the table present volumetric assessment, followed by LA myocardial function. Additionally, LV diastolic and systolic values are presented. Correlations with age and corresponding *P*‐values are given.

Bold means statistically significant correlation with age as a continuous variable.

### 3.2 LA myocardial function

**Figure 3:**
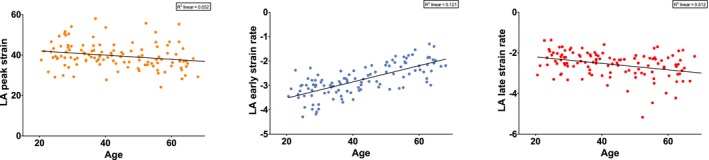
Correlations between left atrial (LA)‐strain, LA‐Sre and LA‐Sra and age. Each dot represents one individual's measurement. The fitted lines and *r* ^2^ values are given. All three variables were significantly correlated with age

**Figure 4:**
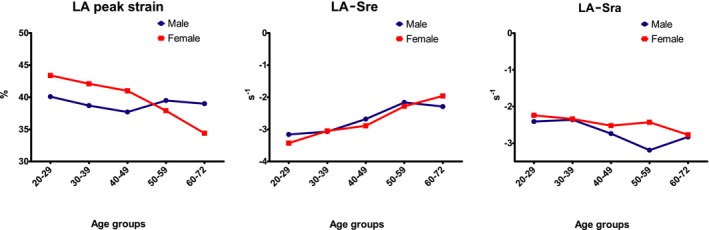
Three graphs showing left atrial myocardial function per sex for each age group

Left atrial‐strain analysis results are shown in Table 4, including the feasibility, which ranged from 78.2% to 80.3%. LA‐strain was lowest in the oldest age groups as was LA‐Sre, but LA‐Sra increased with age (Figure 3). LA‐Sra was significantly more negative in men than women, no sex‐dependent differences were found in LA‐strain and LA‐Sre (Figure 4). The limits of normal (mean ± 2 SD) were also calculated (Table 5).

**Table 5:** Limits of normal for LA function assessed with volumetric and myocardial deformation

	Entire study		20–29 y		30–39 y		40–49 y		50–59 y		60–72 y	
	LLN	ULN	LLN	ULN	LLN	ULN	LLN	ULN	LLN	ULN	LLN	ULN
LA volumetric function												
Total emptying volume (mL/m^2^)	9.1	28.3	9.8	28.6	9.7	27.7	5.7	30.9	10.8	27.2	8.7	27.5
Total emptying fraction (%)	50.5	81.3	54.2	83.4	56.6	76.6	45.9	79.9	49.2	80.4	46.7	76.3
Expansion index (%)	58.4	344	71.5	405.5	113.8	298.6	42.6	325.8	45	353.8	65.7	272.9
Passive emptying volume (mL/m^2^)	2.4	19.2	7.1	19.5	3.7	19.7	1.1	19.5	2.8	17.2	0.3	15.9
Passive emptying fraction (%)	14.5	59.7	34.1	61.7	22	59.6	17.3	52.1	15	52.6	7.8	45.4
Active emptying volume (mL/m^2^)	2.2	13.8	1.3	10.5	3.4	10.6	2	14	3.8	14.2	4.4	15.6
Active emptying fraction (%)	24.2	64.2	19.3	61.3	28.1	58.1	23.7	62.9	26.3	67.1	26.5	67.7
LA myocardial deformation analysis												
LA‐strain (%)	27	52.2	28.7	54.7	29.8	51	27.5	50.7	24.9	52.5	−49.7	−24.5
LA‐Sre (s^−1^)	−4.02	−1.5	−4.37	−2.21	−3.7	−2.42	−3.53	−2.01	−3.13	−1.33	−3.06	−1.26
LA‐Sra (s^−1^)	−3.81	−1.33	−3.37	−1.29	−3.15	−1.55	−3.75	−1.55	−4.43	−1.23	−4.07	−1.55

LLN = lower limit of normal; ULN = upper limit of normal.

### 3.3 Correlations

Besides age, LA reservoir function did not correlate with baseline characteristics (Table 6). Conduit function decreased slightly with increasing weight, BMI, and blood pressure, while pump function increased with BMI, heart rate, and blood pressure. Conduit and pump function correlated well with LV diastolic parameters. LA‐strain, LA‐Sre, and LA‐Sra correlated well with their volumetric counterparts, LA expansion index and passive and active emptying fraction(*r*: 0.471 *P*: <0.001, *r*: −0.613 *P*: <0.001, *r*: −0.541 *P*: <0.001).

**Table 6:** Table describing correlations between LA function (volumetric and myocardial) and baseline characteristics

	LA‐strain	LA‐Sre	LA‐Sra
	*r*	*r*	*r*
Age	−**0**.**227** *	**0**.**715** **	−**0**.**348** **
Height	−0.045	0.011	−0.012
Weight	−0.068	**0**.**246** *	−0.153
Body mass index	−0.043	**0**.**307** *	−**0**.**197** *
Body surface area	−0.067	**0**.**185** *	−0.119
Heart rate	−0.052	0.076	−**0**.**254** *
Systolic blood pressure	−0.081	**0**.**186** *	−**0**.**275** *
Diastolic blood pressure	−0.1	**0**.**333** *	−**0**.**295** *
E‐wave	**0**.**331** **	−**0**.**566** **	**0**.**182** *
A‐wave	−0.004	**0**.**422** **	−**0**.**367** **
E′	**0**.**331** **	−**0**.**697** **	**0**.**268** *
E/e′	−0.111	**0**.**298** *	−0.128
Left atrial expansion index	**0**.**468** **	−**0**.**381** **	−**0**.**257** *
Left atrial passive emptying fraction	**0**.**354** **	−**0**.**590** **	**0**.**249** *
Left atrial active emptying fraction	**0**.**198** *	0.115	−**0**.**545** **
LA‐Sra	−**0**.**478** **	–	–

Bold mean statistically significant, *P‐value < 0.05, **P‐value < 0.001

### 3.4 Reproducibility

Interobserver agreement was assessed for volumetric and strain measurements: Mean difference for LA maximum volume was −5.2 ± 12.1 mL. For pre‐a‐wave and minimum volume, this was −0.9 ± 10.2 and −1.0 ± 8.4 mL, respectively. Regarding strain measurements, mean difference for LA peak strain, early and late strain rate were 1.83 ± 7.91%, −0.04 ± 0.63, and 0.03 ± 0.67 s^−1^, respectively.

## 4 DISCUSSION

This prospective study shows that LA function assessed with volumetric and myocardial methods is feasible in a healthy population and that age and LV diastolic function are important determinants of LA function. This study presents values per age decade for LA volumetric and myocardial function in a healthy population.

The largest body of evidence with regard to LA assessment is on LA maximum volume; this reflects remodeling due to increased LV filling pressures. The upper limit of normal is set at 34 mL/m^2^, regardless of age, though recent studies showed that LA maximum volume increases with age.^3^, ^4^, ^5^, ^21^ This is especially true in the elderly; no correlation was found in our cohort which included individuals up to 72 years old. We speculated that by using STE, LA dysfunction could be detected earlier, which suggests that LV diastolic dysfunction can be detected before apparent LA dilatation, providing clinicians a possibility to intervene earlier. Our results show that LA peak strain did increase with age, which may implicate that strain is a more sensitive marker for LA remodeling in an earlier stage. A recent study also demonstrated that LA myocardial function was diminished in patients with LV diastolic dysfunction while there was no apparent LA dilatation.^19^

### 4.1 LA volumetric vs myocardial function

This study demonstrates that LA volumetric and myocardial assessment is highly feasible. We recognize that the BSA‐indexed maximum volume in our study was large according to current guidelines. However, with parameters such as LA expansion index, passive and active emptying fraction this is no longer relevant, since these measurements are relative.^22^ Therefore, the reference values of LA volumetric and myocardial function can be extrapolated to other populations. However, there are certain disadvantages to volumetric assessment, like the assumption of geometrical shapes and relatively low reproducibility of especially smaller volumes. STE can overcome these shortcomings because strain analysis does not rely on geometrical assumptions.

### 4.2 Factors influencing LA function

There are a lot of factors that could influence LA volume and consequently function. We have assessed the LA through volumetric function with total emptying fraction, a sort of ejection fraction of the LA. It is well known that this is divided into a passive and active phase and that a portion will flow back into the pulmonary veins. Therefore, we also provided LA expansion index, which better describes reservoir function. Instances that influence LA volumes are age, sex, height, and weight. To address these, LA volumes are often indexed using BSA. In our study, we no longer found differences between men and women after correcting for BSA but we did find relatively high values; a quarter of these volunteers had a LA max volume above the upper limit of normal.^1^ This might be explained by the fact that height and weight are not both as important for LA volume. The Dutch are the tallest people in the world^15^ which is why an additional analysis was done correcting for height as done previously by Eshoo et al.^16^ We found no differences when comparing these results with the BSA corrected volumes. The only exception was that LA maximum volume became significantly but weakly correlated with age (*r*: 0.202, *P*: 0.018).

### 4.3 Effects of age and LV diastolic function on LA function

Several studies have looked at possible age‐related effects on LA size and function, with mixed results.^3^, ^4^, ^5^, ^6^, ^23^ The idea that age influences LA function is not new; Benjamin et al^24^ stated that E‐wave velocity decreases while A‐wave velocity increases with advancing age. Our study demonstrates that age influences LA myocardial function. LA‐strain and LA‐Sre are lowest in older subjects while LA‐Sra is higher, which is as expected. This is partly in line with the study of Morris et al,^19^ who implicated as much for LA‐strain measurements. In our study, LA‐Sre and LA‐Sra also changed with age, though the values that we found for LA‐strain were slightly lower than reported earlier.^19^ This may be due to age differences between studies or intervendor differences, as a recent study showed that QLAB10 reports slightly lower values for GLS than other software packages.^25^ The study of Miglioranza et al,^26^ which looked at influences due to age, showed similar effects, though the actual results cannot be compared as the P‐wave was used as onset.

Currently, there is no consensus on how to assess LA phasic function with STE. In this study, we used R‐wave as onset because that would allow extrapolation of our data to patients with atrial fibrillation. There are other recent studies that used either the R‐wave or the P‐wave as onset showing that both these techniques are possible.^19^, ^26^, ^27^ We choose for strain rate to assess LA booster pump function instead of peak strain, as this was found to be superior.^27^, ^28^ Pathan et al^29^ performed a meta‐analysis to formulate normal values for LA function. Reservoir function was 39.4% which corresponds very well with our findings, unfortunately for conduit and pump function, strain instead of strain rate was used, which makes it impossible to compare our findings.

Left ventricular diastolic dysfunction is closely related with LA function, and our results reflect that as well. E‐ and A‐wave velocity correlated well with LA conduit and pump function, regardless of the method used. An increase in LV stiffness leads to a reduction in LA conduit function, which is compensated by an increase in pump function. This can be witnessed by the E/A‐ratio, which inverses with age. This was seen for the LA myocardial function parameters.

### 4.4 Limitations

This was a single‐center study including Dutch Caucasian subjects. Extrapolation to other ethnicities should be done with caution. We used QLAB for the strain analysis, though a recent study found no differences between vendors for LA measurements,^29^ comparison with other vendors should be done with caution. Also, subjects had no restrictions regarding food intake prior to the echocardiographic examination. This could influence tissue‐ and pulsed‐Doppler measurements.^30^

### 4.5 Clinical implications

The results from this study may add to the foundation to formulate reference values regarding LA functional analysis, in preparation for studies to determine potential diagnostic and prognostic value which may eventually be used to assess patients in a clinical setting. In our experience, LA functional analysis, especially myocardial deformation, is easy and quick to perform. As expected, age plays an important role, which is why we propose age‐dependent reference ranges. The fact that LA maximum volume did not correlate with age but LA‐strain did indicates that functional assessment is a more sensitive marker.

Future studies should investigate the potential prognostic value of LA function and which technique, myocardial deformation or volumetric assessment, is most valuable.

## 5 CONCLUSION

Left atrial volumetric and myocardial function measurement is a viable option, and age‐dependent reference ranges for LA phasic function are presented. LA myocardial and volumetric function parameters have proven to be age‐ but not sex‐dependent. Considering the high feasibility and clinical relevance of LA myocardial function measurements, these results can help integrate LA STE analysis into clinical practice.

## CONFLICT OF INTEREST

None declared.

## References

[1] Lang RM , Badano LP , Mor‐Avi V , et al. Recommendations for cardiac chamber quantification by echocardiography in adults: an update from the American Society of Echocardiography and the European Association of Cardiovascular Imaging. Eur Heart J Cardiovasc Imaging. 2015;16:233–270. 25712077 10.1093/ehjci/jev014

[2] Appleton CP , Galloway JM , Gonzalez MS , et al. Estimation of left ventricular filling pressures using two‐dimensional and Doppler echocardiography in adult patients with cardiac disease. Additional value of analyzing left atrial size, left atrial ejection fraction and the difference in duration of pulmonary venous and mitral flow velocity at atrial contraction. J Am Coll Cardiol 1993;22:1972–1982. 8245357 10.1016/0735-1097(93)90787-2

[3] Okamatsu K , Takeuchi M , Nakai H , et al. Effects of aging on left atrial function assessed by two‐dimensional speckle tracking echocardiography. J Am Soc Echocardiogr. 2009;22:70–75. 19131005 10.1016/j.echo.2008.11.006

[4] Nikitin NP , Witte KK , Thackray SD , et al. Effect of age and sex on left atrial morphology and function. Eur J Echocardiogr. 2003;4:36–42. 12565061 10.1053/euje.2002.0611

[5] Boyd AC , Schiller NB , Leung D , et al. Atrial dilation and altered function are mediated by age and diastolic function but not before the eighth decade. JACC Cardiovasc Imaging. 2011;4:234–242. 21414570 10.1016/j.jcmg.2010.11.018

[6] Kou S , Caballero L , Dulgheru R , et al. Echocardiographic reference ranges for normal cardiac chamber size: results from the NORRE study. Eur Heart J Cardiovasc Imaging. 2014;15:680–690. 24451180 10.1093/ehjci/jet284 PMC4402333

[7] Cameli M , Caputo M , Mondillo S , et al. Feasibility and reference values of left atrial longitudinal strain imaging by two‐dimensional speckle tracking. Cardiovasc Ultrasound. 2009;7:6. 19200402 10.1186/1476-7120-7-6 PMC2652427

[8] Vianna‐Pinton R , Moreno CA , Baxter CM , et al. Two‐dimensional speckle‐tracking echocardiography of the left atrium: feasibility and regional contraction and relaxation differences in normal subjects. J Am Soc Echocardiogr. 2009;22:299–305. 19258177 10.1016/j.echo.2008.12.017

[9] Kim DG , Lee KJ , Lee S , et al. Feasibility of two‐dimensional global longitudinal strain and strain rate imaging for the assessment of left atrial function: a study in subjects with a low probability of cardiovascular disease and normal exercise capacity. Echocardiography. 2009;26:1179–1187. 19725856 10.1111/j.1540-8175.2009.00955.x

[10] Saraiva RM , Demirkol S , Buakhamsri A , et al. Left atrial strain measured by two‐dimensional speckle tracking represents a new tool to evaluate left atrial function. J Am Soc Echocardiogr. 2010;23:172–180. 20152699 10.1016/j.echo.2009.11.003

[11] Sun JP , Yang Y , Guo R , et al. Left atrial regional phasic strain, strain rate and velocity by speckle‐tracking echocardiography: normal values and effects of aging in a large group of normal subjects. Int J Cardiol. 2013;168:3473–3479. 23706316 10.1016/j.ijcard.2013.04.167

[12] Xia J , Gao Y , Wang Q , et al. Left atrial function examination of healthy individuals with 2D speckle‐tracking imaging. Exp Ther Med. 2013;5:243–246. 23251276 10.3892/etm.2012.789 PMC3524256

[13] Menting ME , McGhie JS , Koopman LP , et al. Normal myocardial strain values using 2D speckle tracking echocardiography in healthy adults aged 20 to 72 years. Echocardiography. 2016;33:1665–1675. 27550630 10.1111/echo.13323

[14] Rosner A , Barbosa D , Aarsaether E , et al. The influence of frame rate on two‐dimensional speckle‐tracking strain measurements: a study on silico‐simulated models and images recorded in patients. Eur Heart J Cardiovasc Imaging. 2015;16:1137–1147. 25762560 10.1093/ehjci/jev058

[15] Collaboration NCDRF . A century of trends in adult human height. Elife. 2016;5:e13410. 27458798 10.7554/eLife.13410 PMC4961475

[16] Eshoo S , Ross DL , Thomas L . Impact of mild hypertension on left atrial size and function. Circ Cardiovasc Imaging. 2009;2:93–99. 19808574 10.1161/CIRCIMAGING.108.793190

[17] Nagueh SF , Smiseth OA , Appleton CP , et al. Recommendations for the evaluation of left ventricular diastolic function by echocardiography: an update from the American Society of Echocardiography and the European Association of Cardiovascular Imaging. Eur Heart J Cardiovasc Imaging. 2016;17:1321–1360. 27422899 10.1093/ehjci/jew082

[18] Mor‐Avi V , Lang RM , Badano LP , et al. Current and evolving echocardiographic techniques for the quantitative evaluation of cardiac mechanics: ASE/EAE consensus statement on methodology and indications endorsed by the Japanese Society of Echocardiography. Eur J Echocardiogr. 2011;12:167–205. 21385887 10.1093/ejechocard/jer021

[19] Morris DA , Takeuchi M , Krisper M , et al. Normal values and clinical relevance of left atrial myocardial function analysed by speckle‐tracking echocardiography: multicentre study. Eur Heart J Cardiovasc Imaging. 2015;16:364–372. 25368210 10.1093/ehjci/jeu219

[20] Bland JM , Altman DG . Statistical methods for assessing agreement between two methods of clinical measurement. Lancet. 1986;1:307–310. 2868172

[21] D'Andrea A , Riegler L , Rucco MA , et al. Left atrial volume index in healthy subjects: clinical and echocardiographic correlates. Echocardiography. 2013;30:1001–1007. 23594028 10.1111/echo.12217

[22] van Grootel RWJ , Menting ME , McGhie J , et al. Echocardiographic chamber quantification in a healthy Dutch population. Neth Heart J. 2017;25(12):682–690. 29019026 10.1007/s12471-017-1035-7 PMC5691816

[23] Spencer KT , Mor‐Avi V , Gorcsan J 3rd , et al. Effects of aging on left atrial reservoir, conduit, and booster pump function: a multi‐institution acoustic quantification study. Heart. 2001;85:272–277. 11179264 10.1136/heart.85.3.272 PMC1729654

[24] Benjamin EJ , Levy D , Anderson KM , et al. Determinants of Doppler indexes of left ventricular diastolic function in normal subjects (the Framingham Heart Study). Am J Cardiol. 1992;70:508–515. 1642190 10.1016/0002-9149(92)91199-e

[25] Farsalinos KE , Daraban AM , Unlu S , et al. Head‐to‐head comparison of global longitudinal strain measurements among nine different vendors: the EACVI/ASE inter‐vendor comparison study. J Am Soc Echocardiogr. 2015;28:1171–1181, e1172. 26209911 10.1016/j.echo.2015.06.011

[26] Miglioranza MH , Badano LP , Mihaila S , et al. Physiologic determinants of left atrial longitudinal strain: a two‐dimensional speckle‐tracking and three‐dimensional echocardiographic study in healthy volunteers. J Am Soc Echocardiogr. 2016;29:1023–1034. e1023. 27638238 10.1016/j.echo.2016.07.011

[27] Rimbas RC , Mihaila S , Vinereanu D . Sources of variation in assessing left atrial functions by 2D speckle‐tracking echocardiography. Heart Vessels. 2016;31:370–381. 25388354 10.1007/s00380-014-0602-8

[28] Hayashi S , Yamada H , Bando M , et al. Optimal analysis of left atrial strain by speckle tracking echocardiography: P‐wave versus R‐wave trigger. Echocardiography. 2015;32:1241–1249. 25363348 10.1111/echo.12834

[29] Pathan F , D'Elia N , Nolan MT , et al. Normal ranges of left atrial strain by speckle‐tracking echocardiography: a systematic review and meta‐analysis. J Am Soc Echocardiogr. 2017;30:59–70. e58. 28341032 10.1016/j.echo.2016.09.007

[30] Dencker M , Bjorgell O , Hlebowicz J . Effect of food intake on commonly used pulsed Doppler and tissue Doppler measurements. Echocardiography. 2011;28:843–847. 21827540 10.1111/j.1540-8175.2011.01451.x

